# Supplementary material for: Supportive care needs and challenges experienced by women diagnosed with breast cancer in Kumasi, Ghana: A qualitative exploratory study
Source: PLoS One. 2025 Nov 18;20(11):e0336860. doi: 10.1371/journal.pone.0336860 (PMC12626267; doi:10.1371/journal.pone.0336860)
Supplement: S1 File — (DOCX) [file pone.0336860.s001.docx]

**S1: INTERVIEW GUIDE**

**DATA COLLECTION INSTRUMENTS**

This current study titled “**Supportive care needs and challenges experienced by women diagnosed with breast cancer in Kumasi, Ghana**” in which ethical approval has been obtained.

**SECTION A**

**INTRODUCTION AND DEMOGRAPHIC DATA**

How old are you now?

Tell me about your educational background?

What is your Ethnicity?

Kindly let us talk about your religion?

How many chemotherapy sessions have you undergone?

**SECTION B**

**MAIN INTERVIEW SESSION**

What is/are your supportive care needs as you undergo treatment?

***Informational needs***

1. Could you share with me how you receive information regarding your diagnosis?
2. What do you know concerning your diagnosis? (Probes: disease, causes, symptoms, treatment)
3. Can you tell me how you have shared your diagnosis with others?

***Psychological/ Emotional needs***

1. What are your psychological needs as you undergo treatment for this disease? (Interviewer’s note: coping with the disease, self-worth issues, body image disturbance)
2. What emotional issues (anger, despair, fear, hopelessness) do you face during treatment and your emotional needs as you undergo treatment for this condition? (Interviewer’s note: reassurance, comfort, moral support, self-expression)
3. How do you receive psychological and emotional support to cope with the disease?

***Physical/ practical needs***

1. What are your physical/practical needs as you undergo treatment for this disease?
2. What impact does the disease have on your physical and practical needs?
3. How do you receive physical and practical support in dealing with the disease?

***Social needs***

1. What is the effect of this disease on your relationships? *(Probes: relationships with partner, children, family, co-workers, church members and friends).*
2. How are you socially accepted in the community regardless of your condition?

***Spiritual needs***

1. What kind of spiritual support do you need as you receive treatment for this disease?
2. What impact does the disease have on your spiritual life? *(Probes: meaning in life, suffering, pain)*

How do all these supportive care needs help you cope during treatment?

What are the challenges and barriers you encounter in accessing these supportive care needs as you undergo treatment?

Thank you for your time and valuable information on supportive care needs of women diagnosed of breast cancer at Kumasi Metropolis.
