# Supplementary material for: Supportive care needs and challenges experienced by women diagnosed with breast cancer in Kumasi, Ghana: A qualitative exploratory study
Source: PLoS One. 2025 Nov 18;20(11):e0336860. doi: 10.1371/journal.pone.0336860 (PMC12626267; doi:10.1371/journal.pone.0336860)
Supplement: Attachment — Submitted filename: Response to Review.pdf [file pone.0336860.s003.pdf]

University of Ghana
College of Health Sciences
School of Nursing and Midwifery
Department of Adult Health

2nd May, 2025

The Editor
PLOS ONE

Dear Sir/Madam,

RESPONSE TO REVIEW

We thank you for the review of our manuscript titled "Supportive care needs and challenges experienced by women diagnosed with breast cancer in Kumasi, Ghana".

General comments of authors
We have addressed all the concerns of the editorial review in the revised manuscript.

Editorial review
PONE-D-25-23079
Supportive care needs and challenges experienced by women diagnosed with breast cancer in Kumasi, Ghana
Dr Kennedy Dodam Konlan

Dear Dr. Konlan,

We've checked your submission and before we can proceed, we need you to address the following issues:

We noticed that only one page of a two-page ethics approval letter was uploaded. Please upload the complete, original ethics approval letter as required by Question 1 of the Human Subjects Research Checklist.

Additionally, we noted a discrepancy in the reported recruitment dates. On page 5, recruitment is described as occurring in July and August 2023, while on page 9, the dates are listed as between 25th April and 20th May 2023. Could you please clarify which dates are correct and revise the manuscript accordingly prior to resubmission.

We hope you understand the reasons behind this request and look forward to hearing from you.

We've returned your manuscript to your account. Please resolve these issues and resubmit your manuscript within 21 days. If you need more time, please email the journal office at plosone@plos.org. We are happy to grant extensions of up to one month past this due date. If we don't hear from you within 21 days, we will withdraw your manuscript.

Please log on to PLOS Editorial Manager at https://www.editorialmanager.com/pone/ to access your manuscript. You will find your manuscript in the 'Submissions Sent Back to Author' link under the New Submissions menu. Be sure to remove your previous manuscript file if you are uploading a new file in response to these requests. After you've made the changes requested above, please be sure to view and approve the revised PDF after rebuilding the PDF to complete the resubmission process.

We are requesting these changes to comply with the PLOS ONE submission guidelines (https://journals.plos.org/plosone/s/submission-guidelines). Please note that we won't send your

manuscript for review until you have resolved the above requests.

Thank you for submitting your work to PLOS ONE and supporting our mission of Open Science.

Kind regards,

Mia Vanessa Recto
PLOS ONE

Authors' response to editorial review
-We have uploaded the second page of the ethics approval letter as requested by the editor.
-We have clarified the date for the recruitment of the participants as between July 2023 to August 2023 as indicated on page 5 and 9 of the revised manuscript.

We are grateful for the review. Thank you

Yours sincerely,
Dr. Kennedy Dodam Konlan
(Corresponding author)
