# Supplementary material for: Supportive care needs and challenges experienced by women diagnosed with breast cancer in Kumasi, Ghana: A qualitative exploratory study
Source: PLoS One. 2025 Nov 18;20(11):e0336860. doi: 10.1371/journal.pone.0336860 (PMC12626267; doi:10.1371/journal.pone.0336860)
Supplement: Attachment — Submitted filename: Response to reviewers .pdf [file pone.0336860.s005.pdf]

University of Ghana
College of Health Sciences
School of Nursing and Midwifery
Department of Adult Health

29th OCTOBER, 2025

The Editor
PLOS ONE

Dear Sir/Madam,

Response to review

General comments of authors

We have addressed all the comments of the reviewers as suggested and we hope the revised manuscript meets the standards for publications.

Comments of the Reviewers

PONE-D-25-23079R1

Supportive care needs and challenges experienced by women diagnosed with breast cancer in Kumasi, Ghana: A qualitative exploratory study

PLOS ONE

Dear Dr. Konlan,

Thank you for submitting your manuscript to PLOS ONE. After careful consideration, we feel that it has merit but does not fully meet PLOS ONE's publication criteria as it currently stands. Therefore, we invite you to submit a revised version of the manuscript that addresses the points raised during the review process.

==============================

ACADEMIC EDITOR:

The authors should address the comments below to further strengthen their manuscript.

Abstract

1. The phrase “Conclusion: Women diagnosed with breast cancer” should be made “Conclusion: Women diagnosed with BC”.
2. The phrase “integrated into breast cancer care” should be made “integrated into BC care”

Methods

1. Under study design, the authors should kindly indicate why a descriptive exploratory design is appropriate for their study.
2. “Setting” should be revised to “Study setting”
3. Under Study setting, after the abbreviation “KATH” is used, all Komfo Anokye Teaching Hospital should be replaced with the abbreviation.
4. The phrase “at the study site” should be made “study setting” for consistency.
5. Replace the phrase “study site” with “study setting” throughout the manuscript.
6. Under “Selection of participants and data collection”, Komfo Anokye Teaching
7. Hospital should be replaced with the phrase “KATH”.
8. The phrase “not staff at the study settings” should be revised to “not staff at the study setting”
9. The phrase “1st author” should be written as “1st author”
10. The phrase “post bCa diagnosis” should be replaced with “post BC diagnosis”

Results

1. The first three sentences under the result section and the socio-demographic characteristics of the participants sub-section are a repetition. The authors should kindly address it.
2. I suggest the authors delete the statement “The face-to-face semi-structured interviews took place
3. between July, 2023 to August, 2023 and was personally conducted by the researchers.” from the socio-demographic characteristics of the participants sub-section.
4. I suggest the authors merge the information under the sub-section “results from the in-depth interviews” into the sub-themes of “information needs”, “psychological needs”

and “challenges experienced by women post BC diagnosis”. This approach will enhance the flow of the results write-up.

5. The labelling for quote “*“I feel there is the need for better information on the condition so that we can understand what has occurred in our body, I think they should take time to explain more”* is missing.
6. Some of the quotes have full stop at the end, while others do not. I suggest the authors fill all those grammatical errors.
7. In the abstract section, the authors used the abbreviation “BC” for breast cancer. However, in the introduction and discussion sections, the authors used the full word “breast cancer” instead of the abbreviation. Furthermore, in the results section, the authors are using the abbreviation “BC”. I suggest the authors decide on one style and use it throughout the manuscript.
8. The authors used both British and American English. E.g., sub-categorized, decentralized and odour. I suggest the authors decide on one and use it throughout the manuscript.

Discussion

1. I suggest the authors provide a brief statement(s) about the aim of the study before discussing the results of the study.
2. I noticed that the authors had a sub-section “Challenges faced by women diagnosed with breast cancer” under the discussion section. If the authors want to discuss the results in sub-sections, then they should create sub-sections for the two remaining objectives of the study to ensure consistency. Otherwise, they can remove the sub-section for “Challenges faced by women diagnosed with breast cancer”.
3. Under the limitations of the study, the abbreviation for Komfo Anokye Teaching Hospital should be used.
4. The authors have the abbreviation “KBTH”. But the abbreviation was not used in the manuscript.
5. I suggest the authors summarise the write-up under the “ethics approval and consent to participate” sub-section.

:

Authors' Response to Review

Abstract

1. We have done the rephrasing of the conclusion from "Women diagnosed with breast cancer" to : Women diagnosed with BC" in the abstract as suggested by the reviewer. This is found on page 2 of the revised manuscript.
2. We have changed the phrase "integrated into breast cancer care" should be made "integrated into BC care" in the abstract as suggested by the reviewer. This is found on page 2 of the revised manuscript.

Methods

1. We have indicated in the methods section why a descriptive exploratory design is appropriate for their study. This is found on page 4 of the revised manuscript.
2. We have revised the word "Setting" to "Study setting" as suggested by the review. This is on page 4 of the revised manuscript.
3. We have replaced the Komfo Anokye Teaching Hospital with the abbreviation "KATH" after its first usage throughout the revised manuscript.
4. We have replaced the phrase "at the study site" with "study setting" for consistency. This is found on page 6 of the revised manuscript.
5. We have ensured that under "Selection of participants and data collection", Komfo Anokye Teaching Hospital has been replaced with the phrase "KATH". This is found on page 5 of the revised manuscript.
6. We have replaced the phrase "not staff at the study settings" to "not staff at the study setting" as suggested by the reviewer. This is found on page 6 of the revised manuscript.
7. We have re-written the phrase "1st author" to "1st author" in the revised manuscript as suggested by the reviewer.
8. We have re-written the phrase "post bCa diagnosis" to "post BC diagnosis" as suggested by the reviewer.

Results

1. We have removed the repetitions in the results section. This is found on page 9 of the revised manuscript.
2. We have deleted the statement “The face-to-face semi-structured interviews took place between July 2023 to August, 2023 and was personally conducted by the researchers.” from the socio-demographic characteristics of the participants sub-section in the results section as suggested by the reviewer.
3. We have merged the information under the sub-section “results from the in-depth interviews” into the sub-themes of “information needs”, “psychological needs” and “challenges experienced by women post BC diagnosis” to help with information flow. This is found on page 11 of the revised manuscript.
4. We have rectified the labelling for quote “ *“I feel there is the need for better information on the condition so that we can understand what has occurred in our body, I think they should take time to explain more”* which was missing. This is found on page 13 of the revised manuscript.
5. We have rectified the grammatical issues with the quotes as suggested by the reviewer.
6. We have ensured that the abbreviation “BC” for breast cancer used in the abstract has been used throughout the entire work for consistency as suggested by the reviewer.
7. We have corrected the grammatical issues in the manuscript and stuck to British English as suggested by the reviewer.

Discussion

1. We have provided a brief statement about the aim of the study before discussing the results of the study as suggested by the reviewer. This is found on page 20 of the revised manuscript.
2. We have removed the sub-section section “Challenges faced by women diagnosed with breast cancer” in the discussion to ensure the discussion flows consistently as suggested by the reviewer.
3. We have ensured that the abbreviation for Komfo Anokye Teaching Hospital is used in the limitation section on page 24 of the revised manuscript.
4. We have provided where the abbreviation “KBTH” was used in the manuscript. This is found on page 8 of the revised manuscript.

5. We have summarized write-up under the “ethics approval and consent to participate” sub-section as suggested by the reviewer. This is found on page 25 of the revised manuscript

================================

We hope the revised manuscript will meet the standards for publication.

Thank you

Yours sincerely,
Dr. Kennedy Dodam Konlan
(Corresponding author)
