# Supplementary material for: Supportive care needs and challenges experienced by women diagnosed with breast cancer in Kumasi, Ghana: A qualitative exploratory study
Source: PLoS One. 2025 Nov 18;20(11):e0336860. doi: 10.1371/journal.pone.0336860 (PMC12626267; doi:10.1371/journal.pone.0336860)
Supplement: Attachment — Submitted filename: Reviewer report.docx [file pone.0336860.s002.docx]

**Reviewer Report**

Thank you for the opportunity to review this well-written and timely manuscript, which aims to explore the lived experiences and supportive care needs of women with breast cancer in Ghana. The topic is highly relevant, with important implications for patient-centered care in low- and middle-income countries. The paper is generally well-structured and supported by relevant literature. However, I have several suggestions to improve clarity, methodological transparency, and overall rigor.

**Abstract**

1. Please revise the sentence *“The study participants identified the following themes”*. Themes are generated by the research team based on participants’ responses; they are not directly “identified” by participants. Consider rephrasing to indicate that the themes emerged from analysis of participants’ accounts.
2. Briefly state the number of themes and subthemes generated in the study.
3. Replace *“diagnosed of bCa”* with *“diagnosed with breast cancer”* for grammatical accuracy.
4. In the keywords section, only the term **“Keywords”** should be in bold. The list of keywords should not be bolded.

**Introduction**

1. The introduction is well-supported by up-to-date literature.
2. The sentence *“Recent studies [10-14] suggests of a consistent rise in the incidence of breast cancer in Ghana, particularly in the past decade”* contains grammatical errors:
   - “Studies” is plural; use *“suggest”* instead of *“suggests”*.
   - Remove “of” in “suggests of.”
   - Suggested revision: *“Recent studies [10–14] suggest a consistent rise in the incidence of breast cancer in Ghana, particularly in the past decade.”*
3. Clearly articulate the research gap related to supportive care needs of women with breast cancer in Ghana. Some studies have already addressed this so Please emphasize what this study adds to fill the gap and how it advances the understanding of supportive care needs in this context. This and other few studies have been done Akuoko, C. P., Chambers, S., & Yates, P. (2022). *Supportive care needs of women with advanced breast cancer in Ghana*. *European Journal of Oncology Nursing, 58,* 102142. <https://doi.org/10.1016/j.ejon.2022.102142>

**Methods**

1. Provide the full list of inclusion criteria, including age range, diagnostic confirmation, and treatment stage. If certain criteria (e.g., age limits) were not applied, explain why.
2. In the exclusion criteria, clarify what is meant by “biomedical treatment,” as some readers may not be healthcare professionals.
3. Describe in detail how participants were purposively selected, including the recruitment process.
4. Expand on the explanation of data saturation:
   - Describe the criteria used to determine that saturation was reached after the fifteenth interview.
   - Indicate how many women were initially contacted, how many agreed to participate, and how many withdrew after consenting.
5. For the “pre-tested interview guide,” state how many participants it was tested on and whether those pilot participants were included in the final analysis.
6. Specify the location(s) of the interviews, who was present during data collection, and whether any notable incidents or challenges were recorded in the field notes.
7. Clarify whether all authors conducted interviews or whether only certain authors were involved in data collection.
8. Consider formatting the Methods section according to the COREQ (Consolidated Criteria for Reporting Qualitative Research) checklist to ensure completeness and transparency.

**Results**

The results are clearly presented; however, the following additions would enhance the depth of the findings:

1. If available, provide participants’ year of diagnosis, details of their support system (formal or informal), which family members were involved, and the duration of their support.
2. Include marital status and whether participants have children, as these factors may influence supportive care needs.
3. Since financial constraints were a key finding, consider reporting participants’ approximate monthly or annual income ranges for context.
4. The themes “Psychological needs” and “Challenges experienced by women post bCa diagnosis” appear conceptually linked. Consider revising to ensure clear distinction or integration to avoid redundancy.

**Minor Revisions**

- In the Limitations section, correct *“cancerdiagnosis”* to *“cancer diagnosis”*.
- Correct *“KATH- Komfo Anokye Teachigng Hospital”* to *“KATH – Komfo Anokye Teaching Hospital”*.
- Spell out all acronyms at first mention in both the abstract and the main text.

**Overall Assessment:**
The manuscript addresses an important gap in the literature and has potential for high impact. Strengthening the articulation of the research gap, providing more methodological detail, and clarifying certain grammatical points will enhance its rigor and clarity.
